# Intersex Tilapia (*Oreochromis* spp.) from a Contaminated River in Taiwan: A Case Study

**DOI:** 10.3390/toxins1010014

**Published:** 2009-08-13

**Authors:** Peter Lin Sun, Shinn-Shoung Tsai

**Affiliations:** 1 Department of Aquaculture, National Pingtung University of Science and Technology, Nei Pu, Pingtung, 91207, Taiwan; 2 Department of Veterinary Medicine and Southern Taiwan Aquatic Animal Disease Diagnostic Center, National Pingtung University of Science and Technology, Nei Pu, Pingtung, 91207, Taiwan; Email: sstsai@mail.npust.edu.tw

**Keywords:** tilapia, morphological deformity, biomarker, indicator, river pollution, endocrine disrupting chemicals, intersex

## Abstract

River pollution in Taiwan is rather serious, but so far there have been no reports of fish intersex problems. This report reveals that 50% male tilapia in the Era-Jiin River of southern Taiwan were found to be feminized in an October 8, 1994 collection from station EJ-2 of this river. After discounting all other possible causative factors, and correlating with endocrine disrupting chemicals found in this river, we suggest that there is a great possibility that the occurrence of intersex tilapia was caused by these chemicals. The above finding suggests that greater attention needs to be given to endocrine disrupting chemicals problems.

## 1. Introduction

Greater attention is gradually being paid by scientists to the imposex and intersex phenomena of aquatic animals. These phenomena are usually correlated with the presence of endocrine disrupting chemicals (EDCs) in the waters. In gastropods, the imposex phenomenon is very common; but intersex phenomena of fish are less common. Imposex populations of *Hexaplex trunculus* (Gastropoda, Muricidal) from a lagoon in Italy and the coast of Croatia were found due to organotin pollution [1]. In the Gulf of Thailand, imposex was discovered in neogastropods because of tributyltin (TBT) pollution [2]. Along the Portuguese coast, imposex netted whelk *Nassarius reticulates* were found due to organotin contamination [3]. On the coasts of China, imposex of gastropods related to organotin pollution is a widely distributed problem. In total, imposex has been found in over 150 gastropod species [4].

As to intersex in fish, according to a summary of the EDMAR program of the UK, four species of fish (flounder *Platichthys flesus*, viviparous blenny *Zoarces viviparus* and two sand gobies *Pomatoschists minutuus* and *Pom. lozanoi*) in industrialized estuaries showed feminization [5]. Morphological masculinization in female mosquito fish *Gambusia holbrooki* from paper mill effluent contamination was found in Florida, USA [6]. Mosquito fish *G. affinis* all appeared to be males in a small coastal stream in Florida, USA [7]. Intersex was detected in catfish *Clarias gariepinus* in a South African water source with *p*-nonylphenol (*p*-NP) in water and sediments [8]. Young barbell (*Barbus* sp.) captured in the downstream stretch of the Po River were found with intersex gonads [9]. Intersex was discovered in small mouth bass *Micropterus dolomieu* from the Potomac River, West Virginia, USA [10]. In northeastern Germany, the three spined stickleback *Gasterosteus aculeatus* and perch *Perca fluviatilis* collected from freshwater locations, and eelpout *Zoarces viviparus* collected from marine environment displayed intersexuality [11].

In Taiwan, imposex of prosobranch gastropods is commonly observed along the west coast. A survey found that there were four prosobranch gastropods (*i.e.*, *T. clavigera, Nassarius livessens, Babylonia areolata,* and *B. formosel formosae*) that exhibited the imposex phenomenon in intertidal and subtidal areas [12]. Imposex of *T. clavigera* was found to be caused by butyltin and phenyltin pollution [13,14]. Oysters in some parts of Taiwan were found to have 100% masculinization [15]. Imposex was also found in the golden apple snail *Pomacea canaliculata* in Taiwan [16,17], due to large amounts of triphenyltin (TPT) used by agriculture. According to a report by The Taiwan Agriculture Industry Association in 1997, more than 150 metric tons of 45% and 20 metric tons of 2% TPT were used annually for agriculture. Even after this chemical was banned in 1999, 27% of farmers still used TPT in agriculture [17].

Although many gastropods were found to exhibit the imposex phenomenon in Taiwan, so far there are no reports about intersex in fish here. This report describes how we found intersex tilapia from station EJ-2 of the Era-Jiin River, the most contaminated river in Taiwan, during our collection on October 8, 1994. This collection was one of a series of collections for the project “Biomarkers in Fish from Contaminated Rivers of Southern Taiwan”, which was supported by the National Science Council of Taiwan.

In this collection, 18 tilapia were harvested, among which the gonads of nine tilapia were fixed. After histological procedures, the slides of gonads were examined. Results showed that six of nine fish were male, but three of the six males had intersex. Because the intersex phenomenon in fish has also found in Taiwan, in addition to the known imposex gastropods, this may imply that the presence of endocrine disrupting chemical contamination in the Taiwanese environment cannot be neglected.

Now many countries monitor river water quality by routinely examining nutrients (NH_4_^+^-N, NO_2_^-^-N, NO^－^_3_ -N etc.), heavy metals, biological oxygen demand (BOD), chemical oxygen demand (COD), and coliform bacteria, but have not paid enough attention to controlling the endocrine disrupting chemicals contamination problem. This study suggests there is a great possibility that the intersex problem of tilapia at EJ-2 station of Era-Jiin River was caused by endocrine disrupting chemicals, and this morphological change in sexual organ can be used as an environmental contamination indicator for tilapia. 

## 2. Material and Methods

### 2.1. Description of the Sampled River and Collection Stations

Taiwan is narrow and elongated, with high mountains in the middle that extend along the longitudinal axis of the island, and most rivers originate from near the mountain peaks, flow to the ocean down steep slopes, and are rather short, but the Era-Jiin River is considered rather flat, with a slope of only 1:142. The length of the Era-Jiin River is 65.18 km, and the drainage area is 350.4 km^2 ^ [18]. More than 90% of rainfall is recorded during the rainy season from May through October in the southern part of the island [19]. With a flat slope and limited rainfall in the dry season, during the dry season the river flow is rather slow, and pollutants easily accumulate in the riverbed.

**Table 1:** Range of selected heavy metals concentrations of the two Era-Jiin stations in 1993~1996.

	EJ-1	EJ-2
Cd	<0.005 to <0.01	<0.005 to <0.05
Cr	<0.001 to 0.19^*^	<0.001 to 0.29^*^
Cu	0.04 to 0.19^*^	0.01 to 0.22^*^
Pb	0.001 to 0.04	0.01 to 0.111^*^
Zn	0.07 to 0.81^*^	0.6 to 1.0 ^*^
Hg		15.94^a*^

All units are mg/L, except Hg, which is μg/L.

* Indicates that the value exceeds the maximum allowed value.

Source: Environmental Protection Department of Taiwan Provincial Government of years 1994-1997 [25] (except the value with a superscript ^a^).

^a^ Kaohsiung Irrigation Association [26] was the source of the Hg value.

The heavy metal refinery industry along the Era-Jiin River is a major source of pollution. Ranges of heavy metal concentrations at the two collection sites are given in Table 1. From this table we can tell the seriousness of heavy metal pollution in this river, due to the number of metal reclamation activities along the river bank, which include acid washing, electrical wire and cable burning (to burn the wire and extract the metal), and disposal of waste motors. The discharged effluents contain large amounts of heavy metals, as well as polychlorinated biphenyls (PCBs), polychlorinated-dibenzo-*p*-dioxins and dibenzofurans (PCDD/DFs) [20], and dioxin [21]. According to Pan [21], the average value of the total toxicity equivalent quantity (TEQ) (including dioxin and planar polychlorinated biphenyls) of bottom mud samples (dry weight) was 20.9 pg TEQ/g (*n* = 4); among these, the average value of dioxin was 17.8 TEQ/g (*n* = 4), that of planar polychlorinated biphenyls was 3.14 TEQ/g (n = 4). According to Chyan *et al.* [22], after establishment of the metal reclamation industry district along Era-Jiin River in 1996, the long period of burning of electrical wires and cables has caused heavy pollution in the lower section of the river. Ling *et al.* [20] indicated that TEQs of PCDD/DFs and TCDD-EQ of co-PCBs were found in fish from the Era-Jiin River. According to Chang *et al* *.* [23], nonylphenol monoethoxylate (NP1EQ) and nonylphenol (NP) contamination was found in the Era-Jiin River. In addition, di-(2-ethylhexyl) phthalate (DEHP) contamination was also found in the mud of this river [24]. Ranges of endocrine disrupting chemicals found from either mud or water of Era-Jiin River are listed in Table 2.

**Table 2:** Ranges of endocrine disrupting chemicals found from either mud or water of Era-Jiin River.

EDC	Sample	Concentration	References
Dioxin¹	Bottom mud(dry)	0.369~66.9 pg-TEQ/g (Average:20.9 pg-TEQ/g n:4)	[21]
Total PCBs^2^	Bottom mud(dry) (0-5 cm depth)	871-13,615 ng/g	[20]
	Tilapia (dry,5 fish)	502 ng/g	
Di(2.ethylhexyl) phthalate (DEHP) ³	Bottom mud(dry)	0.25 μg/g	[27]
Nonylphenol (NP) ^4^	River water	1.47-12.6 μg/L^2 ^Average:7.01 μg/L	[28]
	Bottom mud(dry)	250-390 μg/Kg dry wt.^3^	

¹ Dioxin samples were collected in 2001 from 4 different stations of Era-Jiin river.

² Total PCBs samples were also collected in 1994, the sampling locations were very close to our sampling site too.

³ DEHP samples were collected from locations close to our sampling location but in 2005.

^4 ^Nonylphenol samples were collected from different locations of Era-Jiin River in 2001.

**Figure 1:**
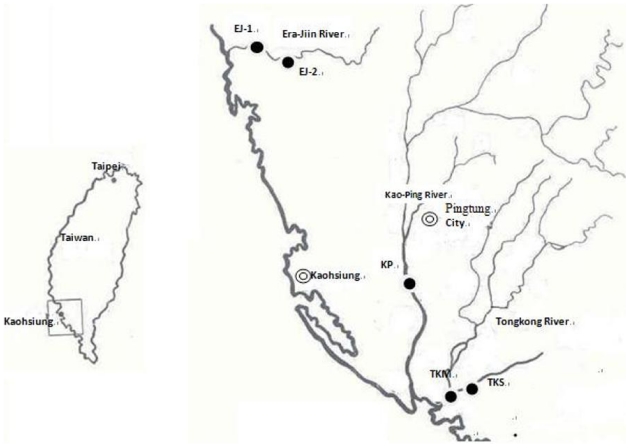
Location of the sampled rivers and sampling stations.

The two stations that tilapia (*Oreochromis* spp.) were collected in this river were EJ-1 and EJ-2. The first station (EJ-1) is a mesohaline station, 6 km upstream of the river mouth, around 200 m wide, with an average depth of 2 m and a mud and sand bottom. The second station (EJ-2) is about 150 m wide, and is a tidal freshwater station, 10.2 km upstream of the river mouth, with an average depth of 1.5 m and a mud bottom. Location of the sampled river and collection station is shown in Figure 1 below.

The sampling stations (EJ-2) is shown in this figure. The sampling stations of some of the other rivers in the project “Biomarkers in Fish from Contaminated River of Southern Taiwan” are also shown in this figure.

### 2.2. Sampling Protocol

On 8 October 1994, tilapia were collected from shallow portions of the river near vegetation using gill nets (with 8-cm stretch mesh) set for no longer than 1 h. In total, 18 tilapia were collected. Fish were examined for morphological abnormalities in the field, weighed, measured, and then sacrificed. Thirteen of the 18 tilapia were randomly selected, and pieces of their organs were removed. Nine of the 13 selected tilapia had mature gonads, and thus were also collected. All organs of each fish were put into a jar, numbered, and fixed immediately in 10% neutral buffered formalin for a minimum of 1 week for the histopathological analysis.

### 2.3. Histological Procedures

All tissues were removed from 10% neutral buffered formalin and put into 60% ethanol (EtOH) prior to standard histological preparation. All tissues were rinsed overnight in running tap water, then put into a tissue processor (Shandon Corporation) and dehydrated through a series of graded ethanol, cleared in xylene substitute, then embedded in paraffin. Paraffin blocks were sectioned at 5 μm, and tissue sections were mounted on slides and stained with Gill’s hematoxylin and eosin (H&E).

## 3. Results

The 18 tilapia collected at station EJ-2 on October 8, 1994 were from serial nos. 17 to 34 of this river. All fish had body and fin lesions and/or necrosis, many fish presented swollen organs and liver color changes. According to histopathological evaluations, there were many histological anomalies in the fish. Taking the examined gill results as an example, 14% of the sampled fish had club lamellae, 28% had fused lamellae, 28% had epitheliocystis, 14% had telangiectasia, and 28% had protozoa. The liver and spleen showed high percentages of macrophage aggregates and granuloma. The above results evidenced that the degraded water quality in this river has caused many pathological changes in fish. The morphological deformities and other abnormalities in tilapia collected in EJ-2 are listed on Table 3. From this table, we can tell those tilapia were inhabiting in very deteriorated environment. As to the three intersex tilapia, besides lesions and necrosis on the body and fins, fish no. 19 (400 g, gonads showed testis) had a pale and swollen liver; no. 32 (294 g) had necrosis of the mouth and a swollen spleen; no. 34 (330 g, gonad showed testis) had necrosis of the mouth, excess mucus secreted by the gills, white spots and a chocolate color of the liver, abdominal dropsy, and swollen kidneys, spleen, and gall bladder. Photos 1, 2, and 3 are testes of the no. 19, 32, and 34 intersex tilapia, respectively. These photos show the feminization of these testes. 

**Table 3:** Morphological deformities and other abnormalities in tilapia collected in EJ-2 station that may be related to poor environmental condition.

Item	Description	Percentage (%) of prevalence	Number of fish discovered among these 18 tilapia
Fin and body surface ^1^	Fin split(s), body surface lesion and/or necrosis	94.4	17
Scale	deformity	16.7	3
Liver	Swollen, discoloration, necrosis, chocolate color	61.1	11
Spleen	Swollen, dark color	44.4	8
Body cavity	Edema, necrosis	22.2	4
Gill	Mucus secretion, necrosis, bleeding, proliferation, pale	27.8	5
Weight(g)	215~400 g		
Other condition ²			

^1 ^In most cases, fish with fin split(s) usually also had body lesion or necrosis.

^2^  Prolepse (1 fish), gall bladder swollen (2 fish), kidney swollen (1 fish), bump-like material on lower jaw (1 fish).

**Figure 2:**
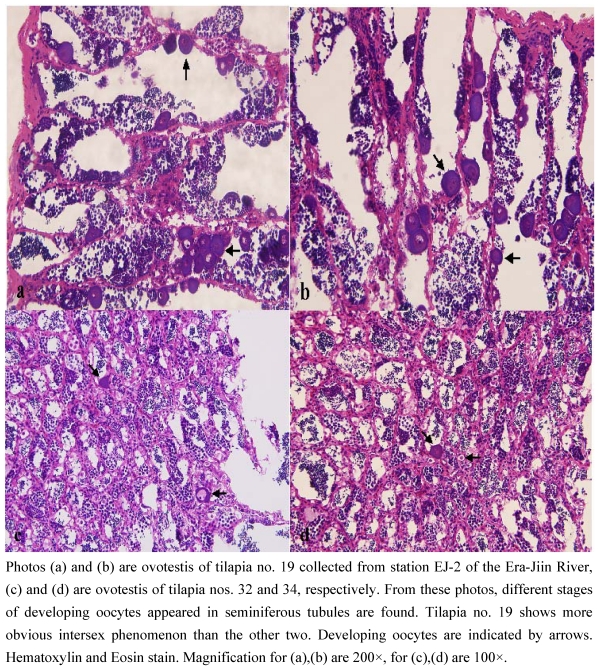
Microphotographs showing intersex occurrence in male tilapia tissues with different degrees of feminization.

## 4. Discussion

Imposex of aquatic animals in Taiwan has been documented for gastropods and some other mollusca, but the intersex phenomenon has not been recorded for fish, and this paper is the first report about this phenomenon in fish from Taiwan. 

Other causative factors that may have caused intersex in tilapia in the Era-Jiin River such as thermal pollution should be discounted, because the section of the river around station EJ-2 has no factories or other sources that discharge substantial amounts of thermal effluents. The possibility of improper hormone-related farmed tilapia escaping from ponds into the river should also be discounted, because hormone-treated tilapia are masculinized for culture purposes, but our findings are of male tilapia becoming feminized. As to the possibility of intersex caused by hybridization, although there no more tilapia were caught after this collection in the Era-Jiin River, more than 700 tilapia were collected from the Kaoping, Tongkong, Linbien, and Suchung Rivers for this project from Autumn 1994 to Spring 1997, but no other intersex tilapia were found. According to the taxonomic identity of the representative tilapia captured in this project by Dr. Marilyn Stiassny of the American Museum of National History, New York, NY, USA, the genetic stock of some of the tilapia that was not pure, but they still could not be considered to be hybrids [29]. Apparently the genetic stock being impure will not yield intersex in tilapia.

The possibility that those collected tilapia had escaped from ponds should also be rejected. Tilapia collected from EJ-1 weighed 54~290 g, but those collected from EJ-2 weighed 215~400 g, and most tilapia collected from EJ-1 had empty stomachs. This implies that the two stations had different ecological backgrounds or even different types of food chains. In addition, according to the taxonomic identity by Dr. Stiassny, the three representative fish from EJ-1 were *Oreochromis mossambicus*, but the three representative fish from EJ-2 were *O. niloticus*, although their genetic stocks were not pure. From these two points, we can tell the fish collected from this river were likely natural inhabitants. Based on the above evaluations, the intersex tilapia collected from EJ-2 were mostly likely caused by endocrine disrupting chemicals, especially when the ratio of intersex was 50%, and these fish were natural inhabitants of this river.

According to documents, the Era-Jiin River is heavily polluted by serious endocrine disrupting chemicals, such as polychlorinated biphenyls (PCBs), dioxin, nonylphenol (NP), nonylphenol monoethoxylate ( NP1EO), and di (2-ethylhexyl) phthalate (DEHP), Hg, and Pb [20,21,22,23,26,27,30].

Some of these endocrine disrupting chemicals, such as PCBs, dioxin, NP, and NP1EO can produce the phenomenon of feminization [31,32]. Among them, NP has estrogenic activity. According to Goksøyr *et al.*, NP can cause ovotestis in laboratory tested fish larvae [33], another study revealed NP can caused gender balance biased toward females (sex ratio = 0.3 male per female) [34]. PCBs have been shown to cause suppression of plasma testosterone in both rainbow trout and carp [35]. Total PCBs in tilapia that were collected in Era-jiin River were 502 and 315 ng/g (dry matter) in two individual samplings, this was an evidence that tilapia were contaminated by this endocrine disrupting chemicals. The fact that most endocrine disrupting chemicals found in the Era-Jiin River can produce feminization may be able to explain why those tilapia collected from EJ-2 had a high ratio (50%) of intersex.
